# Associating (dis)consonance of harmonic intervals with emotional valences: exploring the visual representation of music through crossmodal associations

**DOI:** 10.3389/fpsyg.2026.1744946

**Published:** 2026-06-17

**Authors:** Mayukha Pillay, Tianyi Zhang, Hengyu Li, Charles Spence

**Affiliations:** Crossmodal Research Lab, Department of Experimental Psychology, University of Oxford, Life & Mind Building, Oxford, United Kingdom

**Keywords:** brightness, colour, consonance, emotion, music intervals, roughness

## Abstract

The current paper investigates whether people associate certain emotions and visual features (specifically roughness, colour, and brightness) with different musical intervals. It was hypothesised that more dissonant intervals would be associated more with sadness and tragedy while more consonant intervals would be more strongly associated with happiness and hopefulness instead. It was further hypothesised that the relationship between intervals and emotional valence would be crossmodally mediated by roughness, brightness, and the warmth of colour. We also investigated whether providing an explanation of consonance and dissonance might affect participants’ ratings. A total of 303 UK citizens rated the brightness, roughness, colour warmth, and associated emotions (sadness-happiness; tragic-hopeful) of 13 musical intervals. The results indicated that participants associated more dissonant intervals with sadness and tragedy, with the relationship between the dissonance of intervals and emotional attributes mediated through roughness, warmth of colour, and brightness. Furthermore, those participants who had been provided with an explanation rated the intervals as more dissonant, sadder, and more tragic. The results reveal that the different musical intervals present in musical modes are responsible for the different emotions that are typically associated with certain modes and this relationship can sometimes be mediated by features presented visually.

## 1 Introduction

Music can induce strong emotional responses in listeners (Budd, 1992). People listen to music in order to change, heighten, match, or release different emotions and moods—both as casual listeners and professional musical practitioners (Juslin and Laukka, 2004; Juslin and Västfjäll, 2008; Sloboda and O'Neill, 2001). It is perhaps no surprise, therefore, that understanding music’s ability to elicit emotion has piqued the interest of music-psychology researchers, filmmakers, and those working in marketing (Gabrielsson, 2009; Spence and Keller, 2024). In fact, music-emotion research has been conducted since the Ancient Greek period (Enea, 2023; Young, 1991). One of the fundamental dimensions of music that is known to evoke emotional associations are the musical modes (Carraturo et al., 2025; Gabrielsson, 2009; Hoshino, 1996; Husain et al., 2002; Justus et al., 2018; O’Connell et al., 2025; Perlovsky, 2010; Young, 1991; Zentner et al., 2008).

A musical mode is defined as an organic system (or scale) consisting of pitch sequences arranged in specific orders based on a central note (Hevner, 1935; Schmeling, 2024). Though the specific emotions experienced by listeners vary, most people tend to report similar feelings when they listen to certain musical modes. For example, the Mixolydian mode, often used as background music in video games, has been marketed to create an urge for adventure and a flavour of the mystical (Shimazu, 2023; mostly mentioned in trade publications, e.g., The American Guitar Academy, 2024), with empirical research showing that participants associate the Mixolydian mode with admiration, joy, and serenity^1^ (Straehley and Loebach, 2014). The Dorian mode—generally associated with mystery and holiness and included in many jazz, rock and folk compositions (Carraturo et al., 2025)—is associated with feelings of seriousness, brilliance, constancy, severity, cheerfulness, and virtuousness (Straehley and Loebach, 2014).

Similarly, previous research has also highlighted that changes in musical mode can modulate the emotions that are perceived^2^ and/or experienced in the music, underscoring the role of modes as the cognitive basis of emotional responses to music (D’Onofrio et al., 2020; Straehley and Loebach, 2014; Temperley and Tan, 2012; Webster and Weir, 2005; Young, 1991). It is worth noting that most of the studies that have examined the relationship between emotion and musical modes have tended to explore the different emotions felt. In other words, there is limited research exploring the differences amongst these modes and why different modes result in distinctly different emotional associations (Juslin and Västfjäll, 2008). Therefore, the present study was designed to explore the differences in modes and the emotional attributes that said differences in modes elicit.

### 1.1 Musical structure of modes and intervals

Understanding the different emotions that are associated with modes lies in understanding how these musical modes vary. The seven musical modes (referred to as the diatonic modes) of the Gregorian Musical System^3^ are the Ionian, Dorian, Phrygian, Lydian, Mixolydian, and Aeolian modes. Each of these modes consists of eight notes, with the interval between these notes presenting unique halftone (semitone, defined as the distance between two adjacent tones in a 12-tone scale; Randel, 2003) and/or whole-tone arrangements (two semitones; Randel, 2003) (see Table 1).

**Table 1:** Adopted from Baez (2023), the diatonic modes and scale degrees (the numbers).

Mode	Scale degrees						
Ionian	1	2	3	4	5	6	7
Dorian	1	2	*b*3	4	5	6	*b*7
Phrygian	1	*b*2	*b*3	4	5	*b*6	*b*7
Lydian	1	2	3	*#*4	5	6	7
Mixolydian	1	2	3	4	5	6	*b*7
Aeolian	1	2	*b*3	4	5	*b*6	*b*7

Modes are also often described by flats (*b*) and sharps (#) that show how the intervals (defined as the pitch distance between two notes; Siegel and Siegel, 1977) change when compared to the Ionian mode.

Looking at the composition of the modes shown in Table 1, it can be seen that every mode has a distinctive pattern of half and whole tones (Ramos et al., 2011; Straehley and Loebach, 2014). For illustrative purposes, compare the distinctive patterns of the Dorian and Mixolydian modes. By simply changing the interval of the third scale degree, individuals are more likely to experience feelings of mystery when listening to the Dorian mode (Straehley and Loebach, 2014), compared the feeling of adventure when listening to the Mixolydian mode (Shimazu, 2023). This suggests that the change in intervals between notes is the differentiating factor between the various musical modes and may be responsible for the different emotions that tend to be associated with certain modes. Thus, in the present study, the different intervals and their emotional associations are explored.

The interval is defined as the pitch distance between two notes (Siegel and Siegel, 1977). However, pitch and intervals are perceptual properties of sound, and therefore, understanding their effects on emotional associations may lie in their psychoacoustical or arithmetic properties (Müller, 2015). In short, Pythagoras (6th century BC) theorised that frequency relationships naturally occur between the partials of two or more pitched sounds and their periodicities. These relationships have led to ratios of whole numbers (known as frequency ratios), and these frequency ratios are perceived as intervals. According to Pythagoras, if the frequency ratio between pitched sounds is a fraction of two smaller integers, the interval is said to be consonant (Cazden, 1980). Consonance has also been defined as, “a ‘stable’ sound sensation produced by certain combinations of two tones played simultaneously” (Lots and Stone, 2008, p. 1429). As soon as the frequency ratios become more complex, the sounds are perceived as more dissonant (Cazden, 1980). Dissonance can also be defined as “a combination of tones that clash or create a sense of tension when played together” (John, 2025, p. 1).

Contemporary music tends to follow Pythagoras’s same line of thought, with the most used system referred to as *Just Intonation* ratios (see Table 2; Müller, 2015). From both ratio systems, the minor 2nd (15:16) and the tritone (32:45) have the most complex frequency ratios, thus are classified as the most dissonant intervals. The unison (1:1), octave (1:2) and the perfect 5th (2:3) have the simplest ratios and are perceived as very consonant (Müller, 2015, but for the earliest distinction, see Cooke, 1959). Dissonant intervals are often reported as sounding unpleasant and convey negatively-valanced emotions, with consonant intervals having the opposite associations (Bueno and Ramos, 2007; Costa et al., 2004; Oelmann and Laeng, 2009; Randel, 2003; Mihelač and Povh, 2020). These general findings on consonance and dissonance have primarily appeared in musicology literature (see Di Stefano et al., 2022, 2024 for reviews, or a more recent experiment by Poudrier et al., 2024). Limited empirical research, however, has investigated the effect of specific musical intervals and their emotional associations. Particularly, and to the knowledge of the authors, only Costa et al. (2000, 2004), Oelmann and Laeng (2009), and Straehley and Loebach (2014) have measured perceived or evoked emotion and music using a range of intervals. With the other studies conducted on consonance and/or dissonance, and emotion, by categorising the intervals as either *dissonant* or *consonant*, previous studies provide a general understanding of the effects of consonance and dissonance on the emotional attributes felt by the listener. By doing this categorisation, it is not possible to apply these general responses to deepening the understanding of why emotions are associated with different musical modes. As the distinct structures show, it is the specific intervals and pattern of intervals presented in the modes that differentiate the various musical modes. Therefore, in order to understand the effect of musical modes on emotional response, this study explores the effects of intervals on emotional associations.

**Table 2:** Taken from Müller (2015), the table shows the name of the interval, followed by its difference in semitones (Δ), the interval name, interval notes, just Intonation (JI) ratio and the Pythagorean (Pyt.) tuning ratio.

*Δ*	Interval name	Interval	JI ratio	Pyt. ratio
0	(Perfect) unison	C4 – C4	1:1	1:1
1	Minor second	C4 – D𝑴	15:16	3^5^:2^8^
2	Major second	C4 – D4	8:9	2^3^:3^2^
3	Minor third	C4 – E𝑴	5:6	3^3^:2^5^
4	Major third	C4– E4	4:5	2^6^:3^4^
5	(Perfect) fourth	C4 – F4	3:4	3:2^2^
6	Tritone	C4 – F^#^4	32:45	2^9^:3^6^ or 3^6^:2^10^
7	(Perfect) fifth	C4 – G4	2:3	2:3
8	Minor sixth	C4 – A𝑴	5:8	3^4^:2^7^
9	Major sixth	C4 – A4	3:5	2^4^:3^3^
10	Minor seventh	C4 – B𝑴	5:9	3^2^:2^4^
11	Major seventh	C4 – B4	8:15	2^7^:3^5^
12	(Perfect) octave	C4 – C5	1:2	1:2

### 1.2 Intervals, brightness, roughness and colours

Previous research has documented significant correspondences between music intervals and emotions (Costa et al., 2004; Oelmann and Laeng, 2009). However, there is limited research on how and why an association between musical intervals and emotion exists (Juslin and Västfjäll, 2008). One of the major frameworks discussing how listening to music induces specific emotions was put forward by Juslin and Västfjäll. These researchers suggested that six mechanisms, one of which involves visual imagery, are responsible for eliciting specific musical emotions in the listener. Focusing on visual imagery, the relationship between musical dimensions, visual imagery and emotion association has been discussed in the psychological (including crossmodal research), cognitive, philosophical, historical, educational and the clinical fields for years (Giannos et al., 2021; Küssner et al., 2019). In fact, research alludes to perceived roughness, brightness, and the colour-warmth of music stimuli inducing emotional responses.

#### 1.2.1 Roughness

The perception of roughness has sparked the interest of researchers due to its semantic association in multiple domains. As Di Stefano and Spence (2022, p. 2100) noted: “it seems quite straightforward to observe that the same term, namely roughness, is used in both the visual and auditory domain to describe certain features of sensory stimuli, and this might likely lead to pairings between the two stimuli”. Note that this statement can also be extended to the tactile modality. Indeed, Helmholtz (1877/1954) stated that in the auditory field, roughness is vital in distinguishing the perception of consonance and dissonance. Specifically, Helmholtz refers to roughness as characteristically dissonant, with consonance referred to as the absence of roughness. Dissonant sounds are associated with roughness and sharper, angular shapes (Liew et al., 2017; Ramachandran and Hubbard, 2001). Moreover, sounds with low roughness corresponding with round, soft shapes. Parise and Spence (2012) demonstrated that “rough” sounds (characterised by high-frequency dissonance) were associated with angular shapes, while harmonic (low-frequency and tonal) sounds were linked to round shapes instead. Murari et al. (2015) reported that participants tend to match higher values of visual (images of N 1200 and N 30 sandpapers on a smooth-rough scale) roughness with minor (versus major) tonalities (see Katircilar and Yildirim, 2022, for a similar study with harmonic and inharmonic sounds) Subsequently, Giannos et al. (2021) investigated non-tonal and highly dissonant stimuli and their associations with visual roughness. The results demonstrated that auditory dissonance is perceptually correlated with visual roughness. These studies highlight a relationship between the perception of roughness and consonant/dissonant sounds.

The smoothness/roughness of sounds is also a key feature associated with emotional responses. Rougher and more dissonant sounds, such as human screams and cries (Arnal et al., 2015; Koutseff et al., 2017) and distressed animal calls (Marx et al., 2021; Soltis et al., 2011), signal potential harm or danger to listeners. The roughness of dissonant stimuli can trigger neural synchronisation which, in turn, results in aversive behavioural responses (Arnal et al., 2019; Fishman et al., 2000, 2001). For instance, dissonant sounds have been shown to affect the brain networks associated with fear, pain processing and aversion. This can result in difficulties in focusing on tasks (Arnal et al., 2019). In turn, this has an impact on the aesthetic effect of rough sounds in humans, which tend to be perceived as unpleasant, or at least as less pleasant than relatively smoother sounds (Helmholtz, 1877/1954; see Di Stefano and Spence, 2022, for a review).

The relationship between auditory roughness and aversive responses is similar to the visuotactile perception of roughness, presumably because rough stimuli are more likely to damage one’s skin (Di Stefano and Spence, 2022). This has been supported by research indicating associations between increases in roughness and decreased pleasantness perception (McDermott et al., 2016; Zwicker and Fastl, 2006). Overall, the research shows that roughness is correlated with dissonance, and regardless of the musical system or context, is typically associated with negatively-valanced emotions by listeners. This, then, raises the question of whether roughness perception could mediate the relationship between consonance and/or dissonance, via the different intervals, and their emotional associations (Liew, 2018). The question is further supported by neuroimaging research supporting the existence of roughness-mediated crossmodal associations between auditory and tactile brain networks (see Di Stefano and Spence, 2022, for a review). This neuroimaging research may provide some insight into how some intervals, and thus modes, are associated with different emotions.

#### 1.2.2 Colour and brightness

The use of phrases such as “the blues” and “white noise” suggests perceived associations between aspects of colour (such as the hue category “blue”) and musical dimensions^4^ (such as the genre “Blues”). Relating to the brightness of colour, Marks (1974) investigated the crossmodal association between auditory pitch and visual brightness by matching variations of grey with the pitch of pure tones. The results indicated that the majority of the participants matched increasing pitch to increasing brightness. Collier and Hubbard (2004) reported a relationship between brightness and musical mode. Specifically, the results indicated that ascending minor and major modes were rated as brighter than descending minor and major modes. Within the latter, the descending harmonic minor mode was rated as darker than the descending natural or melodic minor mode. These studies demonstrate a relationship between auditory and visual brightness.

Marks (1978) also found that low-frequency sounds were associated with dark colours and round shapes. Giannakis and Smith (2001) further demonstrated that pitch and loudness was matched to visual hue and visual brightness. Palmer et al. (2016) reported that participants associated highly saturated colours and brightness with faster tempo music in a major mode, and desaturated and darker colours with slow tempo music in a minor mode.^5^ Recently, Harashima et al. (2025) demonstrated that colours of low luminance, low chroma, and purple-red hue were associated with minor chords, whereas high luminance, high chroma, and orange-yellow colours were associated with major chords instead. The studies mentioned above highlight a correlation between musical modes, intervals, and colour/brightness.

Colours (i.e., hues) can trigger emotional responses in people. For example, Birren (1950) reported that warmer hues, such as yellow and red increases arousal more than cooler hues, such as green and blue. This finding has been supported by D’Andrade and Engan (1974), who found that university students associated excitement with red, and playful with yellow. Both findings were supported by Kurt and Osueke (2014), who reported that spaces that were dominantly red induced excitement. It is, however, important to note that many of the earlier studies, including Birren and Kurt and Osueke’s studies, failed to control for brightness or saturation (Wilms and Oberfeld, 2018). This results in some uncertainty as to whether hues are exclusively at play when it comes to the triggering of emotional responses.

The research that has been published to date therefore highlights the existence of relationships between multiple aspects of music, including mode, colours (including the brightness of colours), and emotions (Whiteford et al., 2018, see Spence and Di Stefano, 2022, for a review relating to musical pitch and its effects on evoking emotion). Bresin (2005) explored the matching of colours with the emotion expressed through music. The results indicated that different colours evoked different emotions of the same music. Specifically, the results indicated that red reflected feelings of anger while blue expressed feelings of love. Similarly, Barbiere et al. (2007) documented a relationship between colours such as red, yellow, and green and “happier” classical music, and grey correlated with “sadder” classical music. This relationship was supported by Palmer et al. (2013), who also found that “happier” music was associated with the colour yellow.

Holm et al. (2009) included research on music, colour and emotion to include different musical genres, with the results indicating that black was associated with metal or rock, blue with the blues (the genre), and pink with pop. Hsiao et al. (2017) also demonstrated that the arousal of the music significantly affected the saturation and hue used by professional stage-lighting technicians (see McDonald et al., 2022, for results indicating no significant effect of hue on arousal ratings in the same context). Lindborg and Friberg (2015) found that relatively happier music (as compared to angry, tender, fear-inducing and sad music) was associated with higher levels of lightness, lighter red, and (especially) yellow. Relatively sadder music tended to be associated with darker patches of grey. These studies express the relationship between music, colour, brightness, and emotional response. This relationship provide some support for the suggestion that colour and brightness perception could mediate the relationship between the different intervals and emotional associations.

#### 1.2.3 Visual representations, music, and emotions

The literature discussed above show that intervals and musical modes are associated with roughness, brightness, and aspects of colour perception when indexed visually. The literature also highlights that these visual representations can trigger emotions (just as musical modes do). Therefore, it raises the question of whether music could correspond with emotions associated through these visual representations. According to Küssner and Eerola (2019), some^6^ people may use visual imagery during music listening to regulate their emotional arousal. Belfi (2019) investigated the extent to which emotional valence and arousal, and the vividness of evoked imagery predict the appeal of music. Using a range of different musical genres, the results demonstrated that both emotional valence and the vividness of evoked imagery could predict response to music, however, it is worth noting that there were large inter-individual differences in participants’ judgements. These studies do, however, showcase the relevance of visual imagery to judgements of music aesthetics (at least in sighted individuals), and the possibility that visual imagery is involved in several emotive processes of music listening. Thus, roughness, colour and brightness perception could mediate the relationship between intervals and emotional response.

### 1.3 The present study

This study examines the effects of different musical intervals on associated emotions. To the best of the authors’ knowledge, a limited number of experimental papers have used different intervals (and, thus, varying degrees of dissonance) to measure associated emotions. Furthermore, and as mentioned earlier, of those studies that use musical intervals, the papers use a restricted number of intervals (Costa et al., 2004; Oelmann and Laeng, 2009) or use melodic lines as opposed to harmonic intervals (Straehley and Loebach, 2014). Therefore, this study will use 13 harmonic intervals found in Western musical compositions (and present in Western musical modes). Moreover, the present study also explores an underlying mechanism for the effects of musical intervals on associated emotions by investigating the mediation of visual representations of roughness, brightness, and warmth of colour on the relationship between these two variables. The emotional attributes chosen for this study are sad-happy and tragic-hopeful. This choice of emotions was ascribed to the categorisation of basic (sad-happy) and complex (tragic-hopeful) emotions (Oelmann and Laeng, 2009), with research indicating that basic and complex emotions are perceived differently based on the universality of emotional expressions. Many researchers consider basic emotions to be more innate and universal (due to their having more of a biological function than complex emotions; Juslin, 2013; Hou et al., 2024), with complex emotions receiving more mixed judgements based on cultural context or repeated pairing of said-emotion and music (see Juslin, 2013, for the coding of emotional expression in musical stimuli). Taking all of this into account, the hypotheses are as follows:

*H1:* More dissonant intervals will be associated with sadness and tragedy, whereas more consonant intervals will be associated with happiness and hopefulness instead. Specifically, intervals with more complex frequency ratios will be associated more with sadness and tragedy than other dissonant intervals.

*H2:* The relationship between intervals and emotional attributes will be crossmodally mediated by visual representations of roughness, brightness and the warmth of colour.

Additionally, while providing a definition of consonance and dissonance may affect the dissonance and emotional associations (Aydogan et al., 2018; Emberson, 2016), having a good and clear explanation will likely reduce ambiguity and confusion thus potentially improving the validity of musical interval-related experimental results (Eerola and Vuoskoski, 2013; Luck and Sloboda, 2008). Furthermore, while certain studies provide explanatory accounts of consonance and dissonance (e.g., McLachlan et al., 2013; Plomp and Levelt, 1965) others omit such explanations^7^ (e.g., Komeilipoor et al., 2015; Lahdelma and Eerola, 2016), and no consensus has yet emerged concerning the effects of explaining consonance and dissonance on association behaviour. Thus, in order to explore the effect of explaining musical terminology on emotional ratings, a between-participant variable of *explanation* was added. Note that no previous research has explored the difference in ratings and/or the perception of dissonance and emotional ratings when provided with a clear description of consonance or dissonance (versus not providing an explanation). Therefore, H3 will remain exploratory and non-directional, stating:

*H3:* Providing an explanation of consonance and dissonance will impact the consonance/dissonance and emotional associations.

## 2 Materials and methods

### 2.1 Design and participants

The study followed a 2 (explanation: present, none) by 13 (interval: major 2nd, major 7th, major 6th, major 3rd, minor 2nd, minor 7th, minor 6th, minor 3rd, octave, perfect 5th, perfect 4th, tritone, unison) between-within design, in which, explanation (of consonance/dissonance) was a between-participants factor and interval was a within-participants factor. The dependent variables were the dissonance, colour, brightness, roughness, and emotions associated with the intervals. The musical training sub-scale of the Goldsmiths Music Sophistication Index (Müllensiefen et al., 2014) was included as a covariate in the analysis.

Data were collected from 303 UK citizens (135 female, 165 male, 2 non-binary, 1 did not provide an answer, mean age of 41.40 years, SD = 13.45) through Prolific Academic.^8^ The sample size was determined by an *a priori* analysis. Using G* Power 3.1 (Faul et al., 2007), targeting a small effect size (*f* = 0.30) and a statistical power (1 − *β*) of 0.95 with *α* = 0.05, a sample of 134 people per group was required for the between-subjects factor (150 participants in the consonance/dissonance explanation condition; 153 participants in the no explanation condition). The participants were required to wear head(ear)phones. Inclusion criteria were the following: Normal sense of vision and hearing, English as their first language, and a submission approval rate of 90% or higher on Prolific.

### 2.2 Ethical statement

Ethical approval (MS IDREC) 1,300,708 for this study was obtained from the Medical Science Interdivisional Research Ethics Committee at the University of Oxford in May 2025.

### 2.3 Materials and measures

All interval recordings were created using a MIDI-keyboard and presented as chords with the same starting note (middle C). Interval chords were repeated four times and music samples lasted for exactly 11 s. Samples used in the current study can be found at: OSF | Associating (in)consonance of melodic intervals with emotional valences: Exploring the visual representation of music with crossmodal perception.

A 5-point Likert scale was used to measure dissonance rating (with 1 being consonant, 5 being dissonant). Visual aids adopted from Bonetti and Costa (2017) and Wang et al. (2022) were used to measure roughness and brightness (see Table 3). Emotional ratings were measured by means of two bipolar adjective pairs (*sadness-happiness*, *tragic-hopeful;* Oelmann and Laeng, 2009). The sadness vs. happiness scale used emoticons (variations of frowning and smiling faces, see Table 3). The participants indicated whether the sound was warm- or cool-coloured using a binary forced-choice task (note that the visual aid was taken from Koenderink et al., 2024).

**Table 3:** Questions and scales used in the present study.

Dependent variable	Question	Scale and coding
Dissonance rating	How would you rate the sound?	1 = consonant, 2 = fairly consonant, 3 = neither consonant nor dissonant, 4 = fairly dissonant, 5 = dissonant
Sadness rating	How does this sound make you feel?	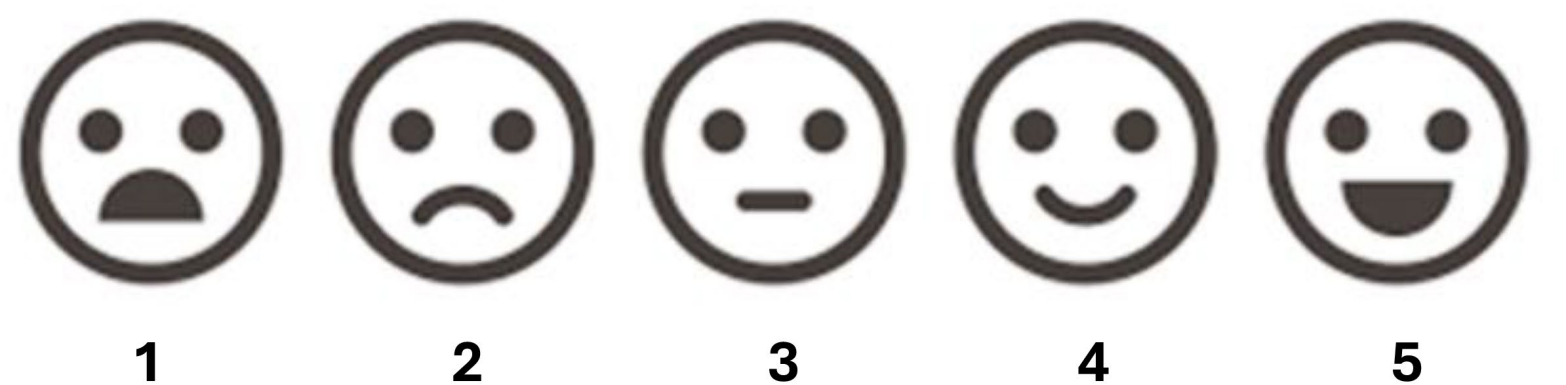
Roughness rating	Rate the roughness of the sound clip	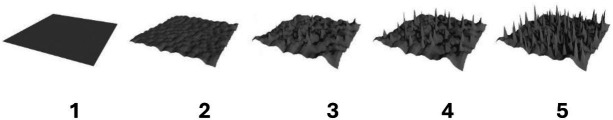
Brightness rating	Rate the brightness of the sound	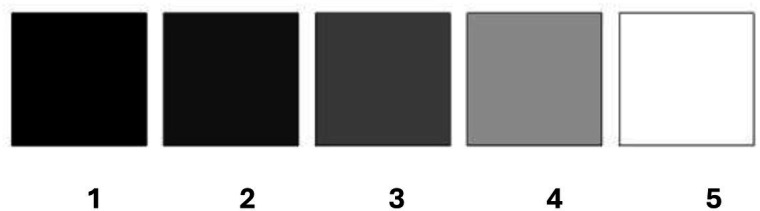
Colour rating	How would you describe the colour of the sound?	Participants chose either warm (1) or cool (0) 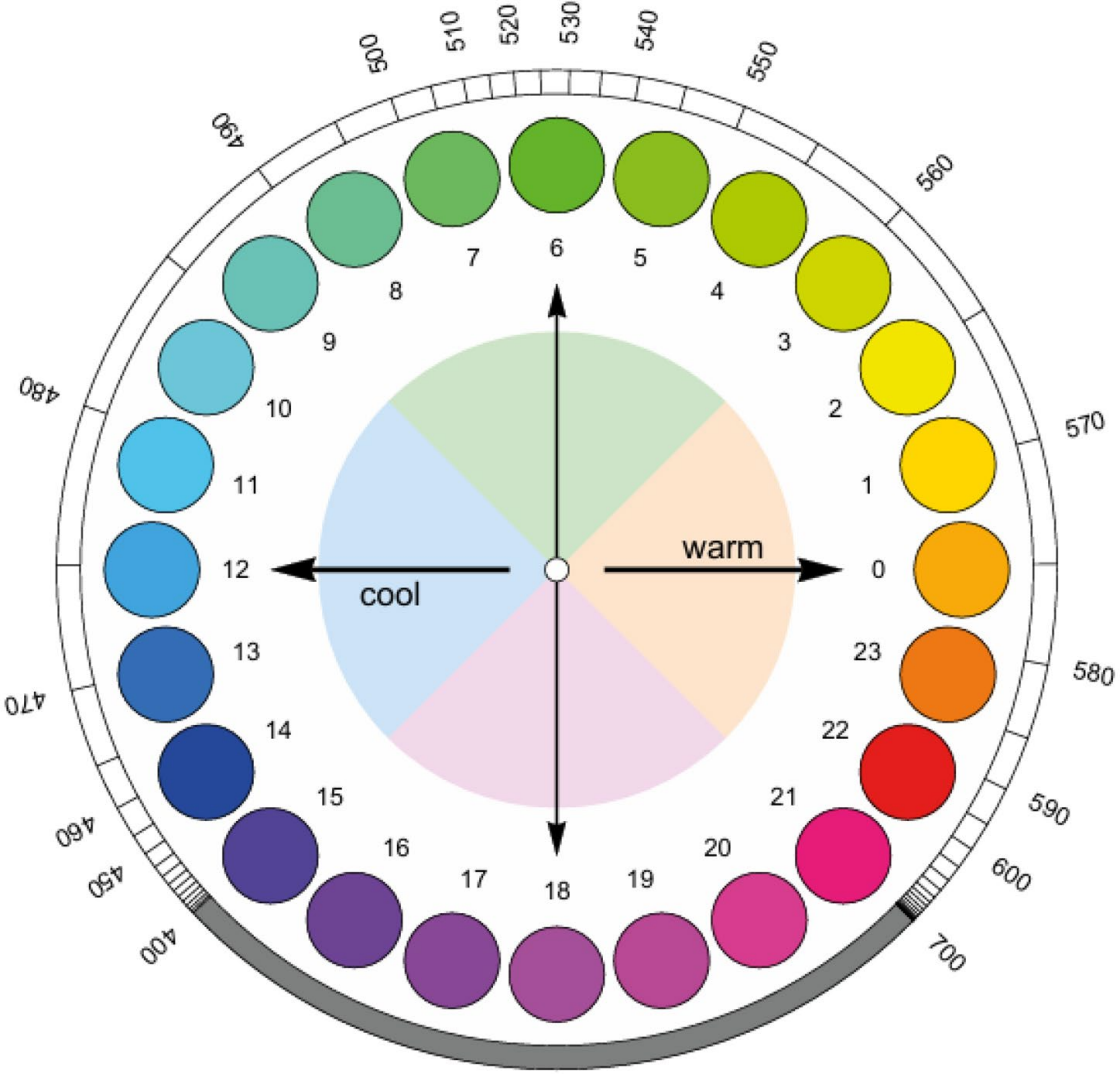
Tragedy/Hopefulness rating	How would you describe the sound clip?	Semantic differential scale with 1 = tragic, and 5 = hopeful
Music training (constructed and coded in accordance with Müllensiefen et al., 2014)	I engage in regular, daily practise of a musical instrument (including voice) for _ years	1 = 0; 2 = 1; 3 = 2; 4 = 3; 5 = 4–5; 6 = 6–9; 7 = 10 or more
	At the peak of my interest, I practised my primary instrument for _ hours per day	1 = 0; 2 = 0.5; 3 = 1; 4 = 1.5; 5 = 2; 6 = 3–4; 7 = 5 or more
	I have never been complimented for my talents as a musical performer (reverse coding)	1 = completely disagree, 2 = strongly disagree, 3 = disagree, 4 = neither agree nor disagree, 5 = agree, 6 = strongly agree, 7 = completely agree
	I have had a formal training in music theory for _ years	1 = 0; 2 = 0.5; 3 = 1; 4 = 2; 5 = 3; 6 = 4–6; 7 = 7 or more
	I can play _ musical instruments	1 = 0; 2 = 1; 3 = 2; 4 = 3; 5 = 4; 6 = 5; 7 = 6 or more
	I would not consider myself a musician (reverse coding)	1 = completely disagree, 2 = strongly disagree, 3 = disagree, 4 = neither agree nor disagree, 5 = agree, 6 = strongly agree, 7 = completely agree

Regarding the individual’s level of music training, the Goldsmiths Music Sophistication Index (Gold-MSI) was used. This is a comprehensive self-reported survey assessing one’s musical sophistication based on their active engagement, perceptual and singing abilities, musical training and emotional response to music (Müllensiefen et al., 2014). The Musical Training factor of the Gold-MSI, consisting of seven questions, was used in the current study (see Table 3).

### 2.4 Procedure

Prior to completing the survey, the participants read through the information sheet and gave their informed consent. The participants were then asked to confirm that they had connected their head(ear)phones and could hear the sound samples at a comfortable listening level. Those participants who received an explanation read the following definitions prior to rating the intervals: “Before completing this survey, it is important for you to understand the terms consonance and dissonance in music theory. Consonance is perceived when tones blend seamlessly, forming a unified auditory image; it’s when musical notes played together sound pleasing and harmonious. Consonant sounds are often described as agreeable and melodious. Dissonance is experienced when tones clash, disrupting the auditory pattern and creating a sense of tension. It can be described as clashing and unresolved.” Those participants who received no explanation proceeded to rating the intervals immediately after adjusting the volume of their headphones. The participants were then presented with a music sample, followed by the following instruction: “Listen to the following sound clip and answer the questions below. You can repeat the sound as often as needed”. The participants rated how dissonant, sad, rough, bright, and tragic the interval was, and what colours (warm vs. cold) they associated with the sounds they heard. The questions were presented in a randomised order to avoid order effects amongst the tested variables. This process was repeated for each interval. The intervals and rating questions were presented in a random order. After that, the participants provided their demographic details concerning age, gender, and nationality, and completed the Gold-MSI. This was followed by a debrief form. The entire study took approximately 13 min to complete.

### 2.5 Data analysis

Jamovi 2.4.8. was used to analyse the data. Multiple generalised linear mixed models (GLMM) were run to test the effects of the thirteen different intervals (within-group factor) and explanation (between-group factor) on dissonance perception, feelings of sadness (vs. happiness) and tragedy (vs. hopefulness), roughness and brightness. Participant ID was the cluster variable and the random intercept. GLMM was used because of the ordinal nature and significant Shapiro–Wilk *p*-values of the data. Colour association was coded as “1 = cool” and “0 = warm” and the effects of intervals and explanation on the colour association were tested using binomial logistic regression. Because of the large number of intervals used (and therefore, the number of pairwise comparisons that were required), post-hoc Bonferroni testing was used to reduce the likelihood of false positives. As musical training may account for people’s understanding and association of musical intervals with emotions (and may reduce some of the error variance of the independent variables), musical training scores were included as a covariate.

To explore how visual representations may explain the underlying connection between intervals and emotional responses, two parallel mediation models were conducted. Both models explored the consonance/dissonance (labelled as dissonance due to the scale used in the survey) perception, with one model exploring sadness-happiness perception (dissonance → roughness/brightness/colour → sadness-happiness) and other exploring tragic-hopefulness perception (dissonance → roughness/brightness/colour → tragic-hopeful scale). Mediation analyses were conducted using lavaan (Rosseel, 2012) in R version 4.4.1 (2024-06-14 ucrt) with 10,000 bootstrapped samples.

## 3 Results

### 3.1 Descriptive results

The detailed means and standard deviations for the dependent variables for the thirteen intervals are in Table 4.

**Table 4:** Means and standard deviations for the dependent variables for the thirteen intervals.

Dependent variables													
Intervals	Major 2nd	Major 7th	Major 6th	Major 3rd	Minor 2nd	Minor 7th	Minor 6th	Minor 3rd	Octave	Perfect 5th	Perfect 4th	Tritone	Unison
Dissonance ratings													
Mean (SD)	3.44 (1.15)	3.49 (1.16)	2.40 (1.07)	2.15 (0.99)	3.94 (1.21)	3.03 (1.11)	2.83 (1.07)	2.86 (1.09)	2.10 (0.98)	2.06 (0.95)	2.36 (1.04)	3.34 (1.19)	2.13 (1.08)
Sadness-happiness ratings													
Mean (SD)	2.49 (0.88)	2.45 (0.91)	3.47 (0.90)	3.63 (0.77)	1.99 (0.96)	2.85 (0.88)	2.90 (0.92)	2.83 (0.86)	3.33 (0.86)	3.51 (0.83)	3.42 (0.83)	2.33 (0.87)	3.21 (0.88)
Tragic-hopeful ratings													
Mean (SD)	2.38 (1.03)	2.35 (1.07)	3.55 (1.10)	3.69 (1.06)	1.77 (0.92)	2.75 (1.11)	2.63 (1.15)	2.61 (1.07)	3.36 (1.13)	3.51 (1.11)	3.38 (1.08)	2.15 (1.06)	3.21 (1.17)
Roughness ratings													
Mean (SD)	2.98 (1.04)	2.95 (1.05)	2.16 (0.91)	2.00 (0.91)	3.61 (1.04)	2.55 (1.01)	2.41 (0.97)	2.46 (0.99)	1.85 (0.90)	1.88 (0.86)	2.05 (0.80)	2.87 (1.09)	1.82 (0.98)
Brightness ratings													
Mean (SD)	2.70 (0.94)	2.84 (1.08)	3.60 (1.06)	3.71 (1.05)	2.23 (1.10)	3.08 (1.04)	3.16 (1.04)	2.95 (0.94)	3.49 (0.99)	3.64 (1.01)	3.39 (0.98)	2.67 (1.04)	3.31 (1.15)
Associated-colour results													
Mean (SD)	0.75 (0.43)	0.72 (0.45)	0.37 (0.48)	0.30 (0.46)	0.84 (0.37)	0.66 (0.47)	0.60 (0.49)	0.70 (0.46)	0.43 (0.50)	0.35 (0.48)	0.46 (0.50)	0.81 (0.38)	0.47 (0.50)

### 3.2 Inferential results

#### 3.2.1 Dissonance rating

The analysis revealed that the overall model was significant (*R*^2^ = 0.33, *p* < 0.001), with a significant main effect of interval (χ^2^ (12) = 1016.27, *p* < 0.001) and explanation (χ^2^ (1) = 6.11, *p* = 0.013). Likewise, the interaction between interval and explanation was significant (χ^2^ (12) = 44.12, *p* < 0.001), and musical training (χ^2^ (1) = 5.61, *p* = 0.018) was a significant covariate. *Post hoc* comparisons revealed that the minor 2nd (*M =* 3.94, SD *=* 1.21) was rated as the most dissonant, followed by the major 7th, major 2nd, and the tritone. By contrast, the perfect 5th (*M =* 2.06, SD *=* 0.95) was rated as the least dissonant interval, followed by the octave, unison, and major 3rd. Providing an explanation (of consonance and dissonance) increased the odds of higher ratings of dissonance across all intervals (*β* = 0.16, *p* = 0.013). Interaction comparisons found that the mean value of dissonance ratings for the major 2nd differed significantly as a function of whether or not an explanation was provided (*p* = 0.022, 95% C.I. = [1.36; 3.32]). Participants with more musical training rated the intervals as more consonant (*β* = −0.16, *p* = 0.018).

#### 3.2.2 Sadness-happiness rating

**Figure 1:**
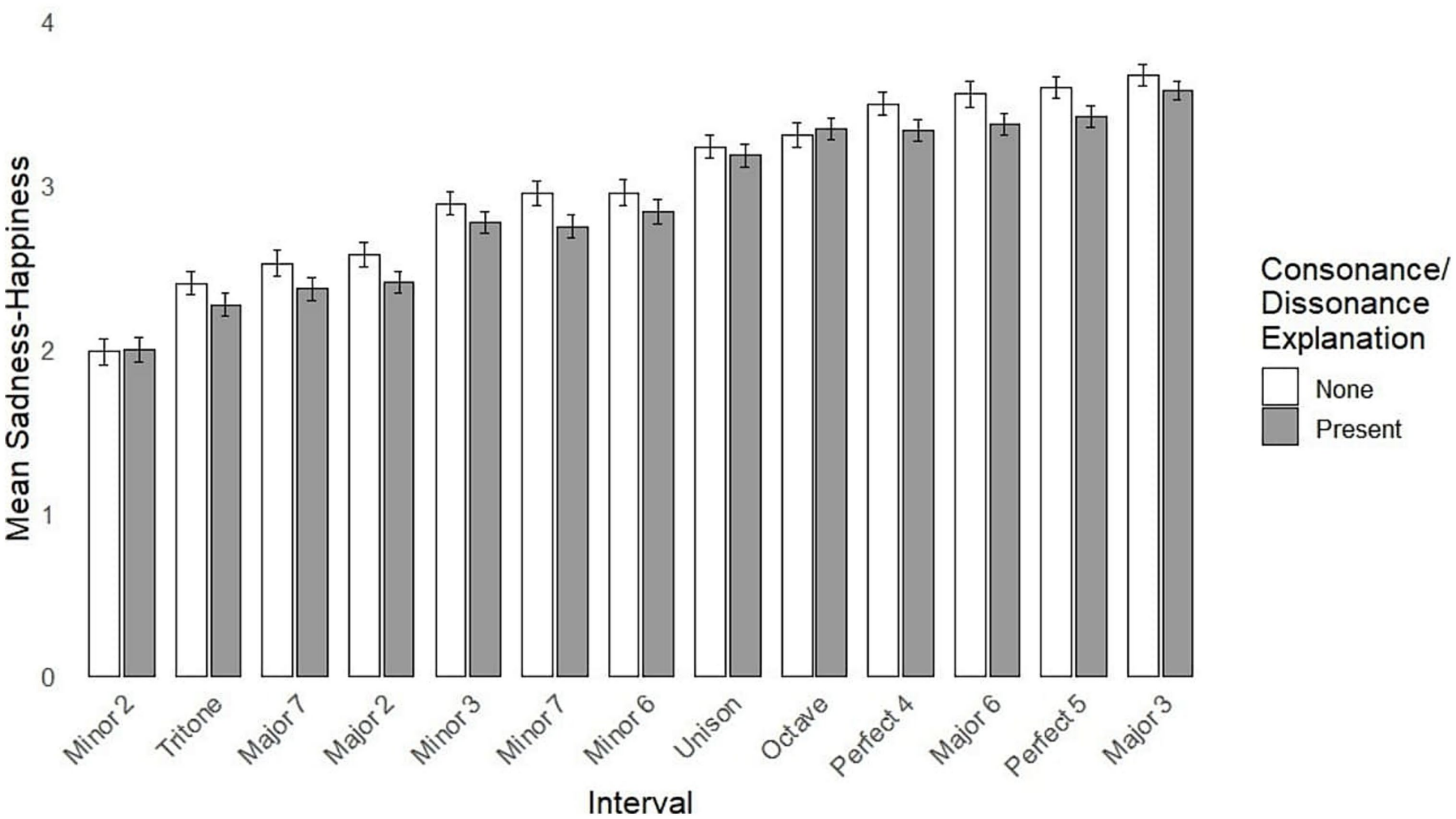
Estimated marginal means of the effects of dissonance on sadness-happiness rating with standard errors (SE) bars.

The analysis revealed that the overall model was significant (*R*^2^ = 0.35, *p* < 0.001), with a main effect of interval (χ^2^ (12) = 1071.71, *p* < 0.001) and explanation (χ^2^ (1) = 31.73, *p* < 0.001). Likewise, musical training was a significant covariate (χ^2^ (1) = 9.36, *p* = 0.002). There was no significant interaction (χ^2^ (12) = 7.54, *p* = 0.820). *Post hoc* comparisons revealed that the minor 2nd was rated as the saddest interval (*M =* 1.99, SD *=* 0.96). This was followed by the tritone, major 7th, and major 2nd. Conversely, the major 3rd (*M =* 3.63, SD *=* 0.77) was rated as the happiest interval, followed by the perfect 5th, major 6th, perfect 4th, and the octave. Furthermore, providing an explanation resulted in participants rating the intervals as sadder (*β* = −0.37, *p* < 0.001), whereas those participants with more musical training rated the intervals as happier (*β* = 0.05, *p* = 0.002) (Figure 1).

#### 3.2.3 Tragic-hopeful rating

**Figure 2:**
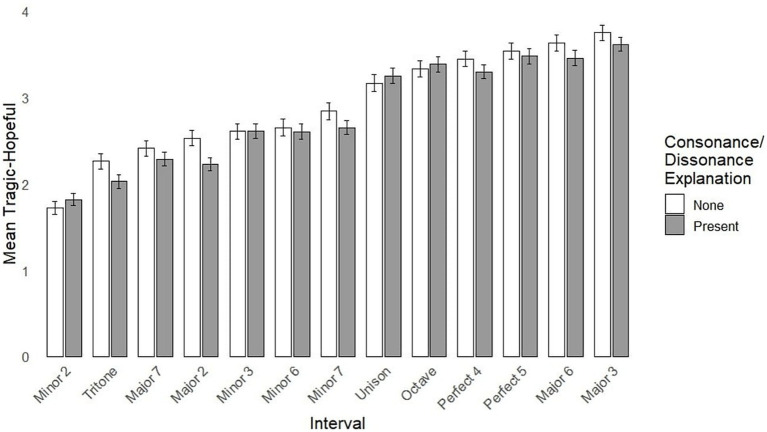
Estimated marginal means of the effects of dissonance on tragic-hopeful rating with standard errors (SE) bars.

The analysis revealed that the overall model was significant (*R*^2^ = 0.33, *p* < 0.001), with a main effect of interval (χ^2^ (12) = 1016.90, *p* < 0.001) and explanation (χ^2^ (1) = 23.1, *p* < 0.001). Likewise, musical training was a significant covariate (χ^2^ (1) = 20.60, *p* < 0.001). There was no significant interaction (χ^2^ (12) = 13.90, *p* = 0.305) between the independent variables. Post hoc comparisons revealed that the minor 2nd (*M =* 1.77, SD *=* 0.92) was rated as the most tragic interval, followed by the tritone, major 7th, and major 2nd. The major 3rd (*M =* 3.69, SD *=* 1.06) was rated as the most hopeful, followed by the major 6th, perfect 5th, and perfect 4th. Furthermore, providing an explanation resulted in participants rating the intervals as more tragic (*β* = −0.31, *p* < 0.001), while those participants with more musical training rated the intervals as more hopeful (*β* = 0.02, *p* < 0.001) (Figure 2).

#### 3.2.4 Roughness rating

The analysis revealed that the overall model was significant (*R*^2^ = 0.34, *p* < 0.001), with a main effect of interval (χ^2^ (12) = 990.26, *p* < 0.001), but not of explanation (χ^2^ (1) = 0.06, *p* = 0.802) nor musical training (χ^2^ (1) = 0.52, *p* = 0.469). There was, however, an interaction between interval and explanation (χ^2^ (12) = 29.14, *p* = 0.004). Post hoc comparisons revealed that the minor 2nd was rated as the roughest interval (*M =* 3.61, SD *=* 1.04), followed by the major 2nd, major 7th, and tritone. By contrast, unison (*M =* 1.82, SD *=* 0.98) was rated as the least rough interval, followed by the octave, perfect 5th, major 3rd and the perfect 4th. Although significant, the pairwise comparisons did not reveal significant effects between the same interval as a function of whether an explanation was provided or not (all Bonferroni *p*-values are 1.00).

#### 3.2.5 Brightness rating

The analysis revealed that the overall model was significant (*R*^2^ = 0.23, *p* < 0.001), with a main effect of interval (χ^2^ (12) = 643.32, *p* < 0.001) but not of explanation (χ^2^ (1) = 0.33, *p* = 0.568), musical training (χ^2^ (1) = 0.74, *p* = 0.389), nor the interaction between interval and explanation (χ^2^ (12) = 11.98, *p* = 0.448). Post hoc comparisons revealed that the major 3rd was rated as the brightest interval (*M =* 3.71, SD *=* 1.05), followed by perfect 5th, major 6th, and octave. Conversely, the minor 2nd was rated as the least bright interval (*M =* 2.23, SD *=* 1.10), followed by the tritone, major 2nd, and major 7th.

#### 3.2.6 Colour-warmth association

The analysis revealed that the overall model was significant (χ^2^ (14) = 539.00, *p* < 0.001) with a main effect of interval (χ^2^ (12) = 529.76, *p* < 0.001) but not of explanation (χ^2^ (1) = 3.43, *p* = 0.064). Similarly, musical training was a significant covariate (χ^2^ (1) = 9.82, *p* = 0.002). The major 3rd was most strongly associated with warm colours (70%), followed by perfect 5th, major 6th, octave, and perfect 4th. By contrast, the minor 2nd was more strongly associated with cool colours (16%). This was followed by the tritone, major 2nd, major 7th, and minor 3rd. Those participants with more musical training rated the intervals as having a warmer tone (*Z* = 3.13, *p* = 0.002).

#### 3.2.7 The relationship between rated dissonance and felt emotions mediated by visual representations

**Figure 3:**
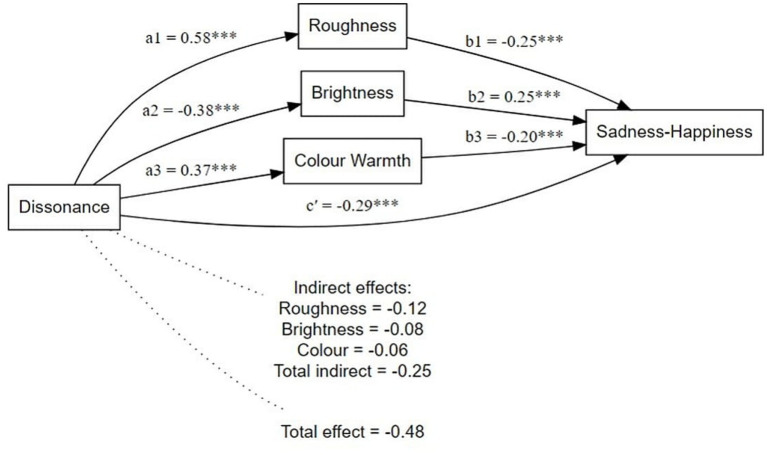
Mediation pathways for the sadness-happiness model. Numbers indicate unstandardized coefficients. *** *p* < 0.001.

The results revealed that the dissonance effect on sadness-happiness ratings is significantly explained by the direct (*c*’ = −16.00, *p* < 0.001, 95% CI [−0.26; −0.20]) relationship between the two variables and indirectly mediated through the visual perceptual qualities (indirect = −26.38, *p* < 0.001, 95% CI [−0.26; −0.23]). The model was significant (*z* = −41.40, *p* < 0.001) and accounts for 49.80% of the variance (Figure 3; Table 5).

**Table 5:** Indirect mediation pathways for the individual mediators in the sadness-happiness model.

Pathway name	Label	*z*	*p*-value	Confidence levels
Indirect roughness pathway	a1.b1	−13.86	*p* < 0.001	95% CI [−0.13; −0.10]
Indirect brightness pathway	a2.b2	−11.92	*p* < 0.001	95% CI [−0.09; −0.06]
Indirect colour pathway	a3.b3	−12.65	*p* < 0.001	95% CI [−0.07; −0.05]

**Figure 4:**
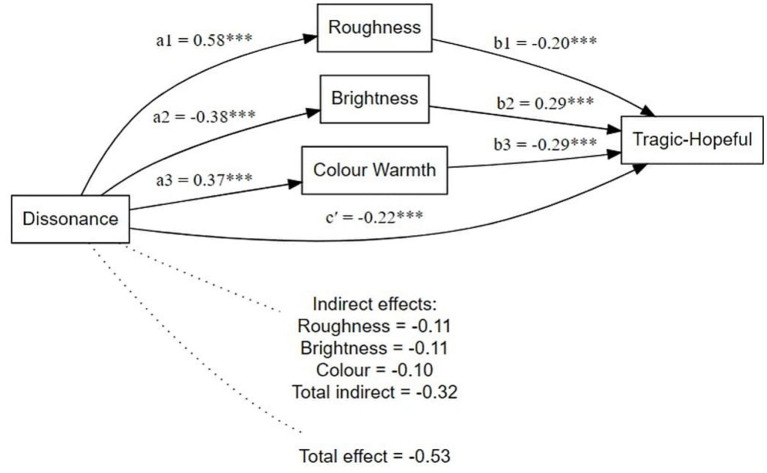
Mediation pathways for the tragic-hopeful model. Numbers indicate unstandardized coefficients. *** *p* < 0.001.

Regarding the tragic-hopeful model, the effect of dissonance ratings on tragic-hopeful ratings is significantly mediated by the visual representation (indirect = − 29.27, *p* < 0.001, 95% CI [−0.34; −0.30]) more than the direct relationship (*c’* = −12.76, *p* < 0.001, 95% CI [−0.24; −0.18]) between dissonance and tragic-hopeful rating. The model was significant (*z* = −37.47, *p* < 0.001) and accounts for 47% of the variance (Figure 4; Table 6).

**Table 6:** Indirect mediation pathways for the individual mediators in the tragic-hopeful model.

Pathway name	Label	*z*	*p*-value	Confidence levels
Indirect roughness	a1.b1	−11.92	*p* < 0.001	95% CI [−0.13; −0.93]
Indirect brightness	a2.b2	−13.22	*p* < 0.001	95% CI [−0.12; −0.90]
Indirect colour	a3.b3	−15.67	*p* < 0.001	95% CI [−0.12; −0.90]

## 4 Discussion

Music evokes emotions, and this has made the artform an important tool for those working in the music, media, film, and marketing industries. While music-psychologists have long explored the way in which musical modes induce specific emotions in the listener, there is limited empirical evidence concerning how and why it is that people reliably associate these Georgian musical modes with certain emotions. The present study was therefore designed in order to explore musical intervals, the structural differentiator between the diatonic modes, and the crossmodal mediation of visual representations in associating emotions (both complex and basic).

The results reported here indicate that different intervals are associated with different emotional responses and concepts represented visually. For instance, the minor 2nd was rated as the most dissonant, saddest, tragic, roughest interval, and was more strongly associated with cool colours. The minor 2nd was also rated as the darkest interval. Indeed, the Locrian mode has the most minor 2nd stepwise motion, which may lead to its association with evil and tension (Ramos et al., 2011).^9^ Conversely, the major 3rd was rated as the most hopeful, happiest, brightest, and likely to be associated with warm colours. Furthermore, the perfect 5th was rated as the most consonant interval, and the unison as the smoothest interval. The Ionian mode has the highest proportion of major and perfect intervals, with Pankhurst (2025) claiming that the Ionian mode is synonymous with feelings of happiness and light.^10^

Looking at the main pattern of results, it is important to note that participants’ dissonant and consonant ratings of intervals match the categorisations that have been suggested previously by mathematicians and psychoacousticians (Müller, 2015). That is, 2nds, 7ths, and tritones are all perceived as more dissonant, while perfect intervals are perceived as more consonant. This is important, as it provides empirical evidence for the connection between the physical nature and the perception of sound (Young, 1991). It is, however, important to note that providing an explanation affected the results, as those participants who were given an explanation rated all intervals as sounding more dissonant than those who were given no such explanation. However, between the two groups (the explanation presented versus no explanation), the general trends were similar, with the only interval being affected significantly by this explanation was the major 2nd.

The results reported here therefore support H1, as dissonant intervals were associated with sadness and tragedy, while consonant intervals were associated with happiness and hopefulness. Additionally, the minor 2nd, which has the most complex frequency ratio (15:16), was perceived as the saddest and most tragic interval, thus supporting the latter of the first hypothesis (that intervals with more complex frequency ratios will be more strongly associated with sadness and tragedy than other dissonant intervals). These findings also coincide with the previous literature on consonance and/or dissonance intervals and the conveying of emotion, thus reinforcing and confirming the conclusion that dissonant intervals are more associated negative-valanced emotions, while consonant intervals express positively-valanced emotions (Costa et al., 2004; Oelmann and Laeng, 2009; Randel, 2003).

Table 7 summarises the main results discussed above. Taking a closer look within the consonant and dissonant responses, a notable characteristic is the clear pattern amongst the dissonant intervals. The most dissonant interval (the minor 2nd) is also the roughest, darkest, most tragic and saddest interval. Conversely, the most consonant interval (the perfect 5th) was not rated as the smoothest (unison) interval, and neither of these intervals were rated as the most hopeful, happiest or brightest (major 3rd). Research has demonstrated that dissonance (compared to consonance) is more significant biologically-speaking in terms of signalling negative emotion (Di Stefano and Spence, 2022). This could be taken to suggest that the response to dissonant intervals could be more innately-determined, and/or automatic, than consonant intervals, resulting in a more salient response to dissonance, whereas the relationship between consonance and emotional ratings could be influenced (or based) on another explanation.

**Table 7:** Characteristic profile of the most significant results.

Interval	Characteristics
Minor 2nd	The most dissonant, saddest, tragic, roughest and likely to be associated with cool colours. Rated as the least bright (darkest)
Major 3rd	Most hopeful, happiest, brightest and likely to be associated with warm colours
Perfect 5th	Least dissonant (most consonant) interval
Unison	Least rough (smoothest) interval

The emotional association with consonant intervals may potentially be explained by the effects of musical training. Participants with more musical training rated all of the intervals as happier, more hopeful, and more associated with warm colours. This partly supports Straehley and Loebach’s (2014) findings, showing that more musically-experienced participants select positive emotions (such as joy and serenity) more frequently for certain modes than those with less musical experience. This could suggest that some aspect of training or familiarity (Di Stefano et al., 2022; McDermott et al., 2010; Popescu et al., 2019) influences the relationship between the consonant intervals and associated emotions. Therefore, more research is required to explore the effects of consonant intervals on emotional attributes.

The present results show that providing an explanation of consonance and dissonance impacted dissonance and emotional ratings. Specifically, participants who were provided with an explanation rated all intervals as more dissonant, sadder, and more tragic. This supports H3, which stated that providing an explanation would affect dissonance and emotional rating scores. Given that the present study is the first paper to explicitly explore the effect of an explanation on dissonance and emotion ratings, more research is required to understand this effect.

The mediation analyses indicate that the relationship between intervals and emotional associations is sometimes mediated crossmodally by roughness, brightness, and colour warmth (when displayed visually), thus supporting Hypothesis 2. Specifically, intervals that were rated as being more dissonant were also associated with higher roughness, lower brightness levels and cool-coloured tones, and evoked more sadness and feelings of tragedy. This is in line with previous research, demonstrating roughness perception as being crucial to the emotional response (specifically pleasantness perception) of sound (Arnal et al., 2019; Helmholtz, 1877/1954; see Di Stefano and Spence, 2022, for a review). Colours with higher luminance, as well as visual stimuli that are brighter and warmer (such as yellow^11^ and orange) tend to be associated with major (perceived as more consonant) chords (Harashima et al., 2025). These colours are categorised as happier (Lindborg and Friberg, 2015; Palmer et al., 2013), induce more excitement (Barbiere et al., 2007; Birren, 1950) and are also rated as more playful (D’Andrade and Engan, 1974).

There are slight differences in the mediations of the emotional attributes. In particular, dissonance has a stronger direct effect on sadness-happiness ratings than on tragedy-hopefulness, with tragic-hopefulness having a stronger reliance on the mediators (than sadness-happiness perception). This result could imply that the association of complex and basic emotions are processed differently through musical intervals. However, given that this study only looks at one basic and one complex emotion, generalisations cannot be made. Future research should therefore explore this further, for this may provide a more nuanced insight into the emotion-associating aspect of music.

The mediation models used accounts for just under 50% of the variance. As mentioned earlier, Juslin and Västfjäll (2008) put forward a framework in which music listening induces emotions via one of six different mechanisms. While one route involves visual imagery, the other five include brain stem reflexes, evaluative conditioning, emotional contagion, episodic memory, and musical expectancy. Investigating the other possible underlying mechanisms and approaching the topic at hand from a neurological^12^ or biological perspective, can increase our understanding of how emotions are associated with music. Similarly, Pimentel et al. (2025) recently examined the matching of scent imagery to diatonic modes. Pimentel and colleagues’ study highlights a relationship between auditory and olfactory crossmodal correspondences. Coupled with research showing the crossmodal matching of colours to olfactory stimuli (see Spence, 2020, for a review), future investigations could therefore explore multisensory imagery effects, looking at crossmodal mediations in multiple senses and how this can influence the emotions associated with musical modes.

The present study is, however, not without its limitations: The focus was on harmonic intervals only and thus no melodic musical intervals were assessed (Kaygusuz and Zuluaga, 2018). Future research should therefore explore the effects of sequential/melodic intervals (i.e., playing the notes one after the other, instead of simultaneously). Note that the study also restricts colour dichotomously to either *warm* or *cool* tones. Relevant here, previous studies have found differences between red and yellow hues (D’Andrade and Engan, 1974; Kurt and Osueke, 2014), both of which are classed as *warm* colour tones. Future research should therefore expand on this concept and explore colours of varying characteristics (such as variations in saturation) to gain further insight into the effects of colour perception on the relationship between musical intervals and emotional associations. This study was restricted to those participants living in the United Kingdom. This was deemed appropriate given the usage of Western modes. Subsequent studies should include different cultures (Miaher, 1976) and/or explore different intervals expressed in other musical cultures, such as microtonal music (Ader, 2020). Similarly, other specific populations, such as those who are congenitally blind or synaesthetes (especially since this study did not specifically exclude colour-music synaesthetes^13^), could provide alternative explanations and insights into the relationship explored in this study. Concurrently, individual differences (beyond musical training) can play a role in felt emotions through music, such as personality (Kallinen and Ravaja, 2006; McDermott et al., 2010). Thus, upcoming investigations should explore the moderating effect of individual differences and expand this idea beyond intervals.

## Data Availability

The raw data supporting the conclusions of this article will be made available by the authors upon request.

## Footnotes

1 For Mixolydian, less musically-experienced participants selected joy, serenity, anticipation, trust, acceptance, and interest equally often. More musically experienced participants selected the same emotions but selected joy and serenity most often, showing an effect of musical training on emotional perception of modes (Straehley and Loebach, 2014).

2 ‘Emotions perceived’ in this context refers both to the emotions expressed by the composer and the emotions that are felt by the listener (see Schubert, 2013, for a review).

3 The Georgian Musical System is the most detailed system of mode classification, originating from the period when Western chant was popular in the Middle Ages (9th and 10th centuries).

4 See Hupé and Dojat (2015) and Havsteen-Franklin and Perboni (2025) for reviews of colour-music synaesthesia.

5 In a workshop, Moss and Bravo (as cited by Eerola, 2017) investigated the matching of brightness, colour saturation, and contrast of Kandinsky paintings to dissonant and consonant intervals. The anecdotal results revealed that consonant sounds, versus dissonant sounds, correlated with brighter colours and sharper contrast.

6 Note the word *some* as there is limited research on blind or aphantasic participants in this context (Jin et al., 2024; Keogh and Pearson, 2018).

7 Note that these studies omit explanations of consonance and dissonance despite these papers exploring the effects of consonance and dissonance.

8
https://www.prolific.com/

9 For example, a famous composition in the Locrian mode is John Kirikpatrick’s *Dust to Dust* (Kirikpatrick, 1971). The piece concerned itself with the inevitability of death from the perspective of a gravedigger, and the emotional tragedy and sombre tone behind the message are enhanced and induced through the piece’s use of the harmonic minor 2 integral to the Locrian mode.

10 Fox’s (2015) main theme from the UNDERTALE soundtrack famously uses the Ionian mode to convey and enhance feelings of optimism.

11 Interestingly, Jonauskaite et al. (2019) conducted a study on crossmodal correspondences between emotion and colour across 55 countries. The researchers found that yellow is more joyful in colder and rainier countries. Thus, further research could explore the relationship between the colour yellow, feelings of joy, and musical intervals in a cross-cultural context.

12 This approach should include the use of neuroimaging and computational modelling methods. Indeed, many papers have approached the question using behavioural measures, and thus, a different perspective could potentially provide new evidence and help researchers to understand the effects of musical intervals on perceived emotions (see Bogert et al., 2016).

13 Note that this is a rare form of synaesthesia (see Curwen, 2018, for a review).
