# Supplementary material for: Associating (dis)consonance of harmonic intervals with emotional valences: exploring the visual representation of music through crossmodal associations
Source: Front Psychol. 2026 Jun 17;17:1744946. doi: 10.3389/fpsyg.2026.1744946 (PMC13320663; doi:10.3389/fpsyg.2026.1744946)
Supplement: Supplementary file 1 [file Supplementary_file_1.docx]

**APPENDIX**

*Solar and lunar metaphorical association with musical intervals*

Solar and lunar metaphorical associations are included here to avoid overcomplicating the main text. The analysis revealed that the overall model was significant (χ² (52) = 704*, p* < .001), with a main effect of interval (χ² (24) = 279.60, *p* < .001) but not of explanation (χ² (2) = 1.98, *p* = .371), musical training (χ² (2) = 4.25, *p* = .119), nor an interaction effect (χ² (24) = 34.57, *p* = .075) between interval and explanation. Post hoc comparisons revealed that the minor 2^nd^ was rated as the most likely to represent the full moon (*probability* = .54, *SE* = .03), the major 2^nd^ was the most strongly associated interval with the half-moon (*probability* = .58, *SE* = .03), and the major 3^rd^, the most strongly associated with the sun (*probability* = .51*, SE* = .03).

**
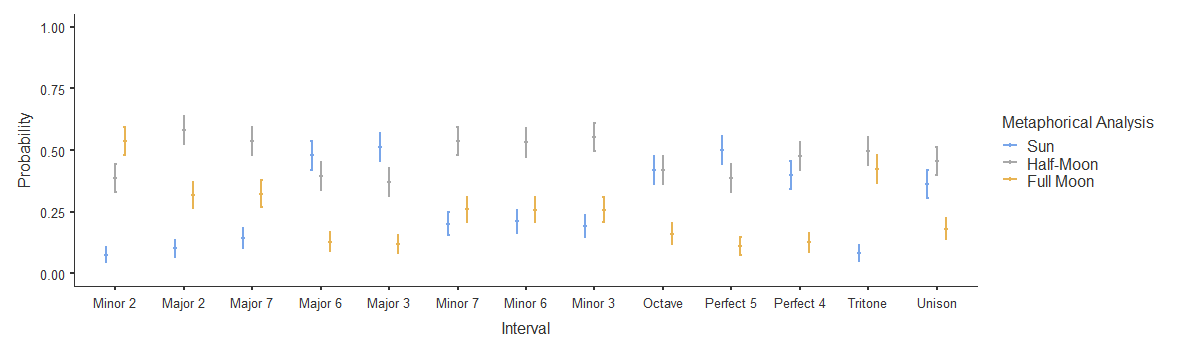
*Figure A1.*** *Estimated marginal means of the effects of intervals on metaphorical analysis and standard error bars*

These results indicate that music composed of 2^nd^s, both major and minor, is associated with the moon. Conversely, the major 3^rd^ was associated with the sun. These results are supported by Olemann and Laeng’s (2008), Experiment 1, showing that the major 2^nd^ was associated most strongly with the moon while the major 3^rd^ was associated with the sun. This suggests that music reflecting solar and lunar themes should consist of major (and minor) 2^nd^s and major 3^rd^s, respectively.

A key example of the use of harmonic 2^nd^s to represent lunar metaphors in music composition is Arnold Schoenberg’s suite *Pierrot lunaire* (*‘*Moonstruck Pierrot’ or ‘Pierrot in the moonlight’, 1912, as cited by Gorshin, 2021). The atonal (meaning not composed in any musical mode or key; Budrys & Ambrazevičius, 2008) nature of the suite results in the frequent use of minor 2^nd^s and major 7^th^s (Symth, 1980) throughout the movements. For illustrative purposes, the seventeenth movement (‘Parodie’) predominantly features harmonic minor 2^nd^s, major 2^nd^s, and major 7^th^s, with the transition from ‘Parodie’ to the eighteenth movement (‘Der Mondfleck’) concluding with an array of descending major 2^nd^s (Gorshin, 2021; McCormick, 2019). Similarly, George Crumb’s *Night of the four moons* (1969, as cited by Chatman, 1974) predominantly uses octaves, major 9^th^s, unisons, and major 2^nd^s to portray the desire to leave the moon untouched by humans (Chatman, 1974, p. 216). Note that while these two examples reflect the use of major and minor 2^nd^s to express moon imagery in musical compositions, no other compositions could be found that  predominantly feature the 2^nd^ harmonic musical intervals, suggesting that minor and major 2^nd^s may be linked more strongly to other themes (as seen with Kirkpatrick’s *Dust to dust* representing death in the main analysis). Further investigations are required to explore the use of harmonic intervals to convey thematic associations and emotional attributes.

While other famous musical pieces that reference the moon [for example, Beethoven’s *Moonlight Sonata*^[[1]](#footnote-1)^ (1801, as cited by Reed, 2025) or Debussy’s *Clair de lune* (1890, as cited by Davis, 2019)] do have some harmonic 2^nd^ intervals present (Chen, 2025), the composers use different musical dimensions (such a rhythmic patterns, timbre, or articulation) to convey these visual concepts. Likewise, Marks’s (1982) study demonstrated empirically that the word *sunlight* was rated as metaphorically louder than the word *moonlight.* Thus, future research could explore the effects of other musical dimensions, including rhythm or volume, on solar and lunar metaphorical associations.

**References**

Budrys, R., & Ambrazevičius, R. (2008). ‘Tonal’ vs ‘atonal’: Perception and tonal hierarchies. In *Proceedings of the 4^th^ Conference on Interdisciplinary Musicology* (pp. 36–37).

Chatman, S. (1974). “Night of the four moons” the elements of sound. *Journal of Musicology Research, 1*(3), 215–223. <https://doi.org/10.1080/01411897408574472>

Davis, E. (2019) Debussy’s Clair de Lune: The romantic piano piece that even has a starring role in Twilight. *Classic fM*, *February 27*. <https://www.classicfm.com/composers/debussy/clair-de-lune-piano-twilight/#:~:text=Claude%20Debussy%20started%20writing%20the%20incredibly%20romantic%20piano,actually%20part%20of%20the%20four-movement%20work%20Suite%20Bergamasque> (accessed [Nov 11, 2025]).

Gorshin, M. (2021). Analysis of ‘Pierrot lunaire’. *Infinite Ocean, May 17.* <https://mawrgorshin.com/2021/05/17/analysis-of-pierrot-lunaire/#:~:text=The%20piece%20begins%20with%20a,effect%20of%20high-pitched%20speaking.> (accessed [Nov 11, 2025]).

Kerney, A. (2017). Moonlight sonata by Beethoven: An analysis. *Piano TV, April 25.* <https://www.pianotv.net/2017/04/moonlight-sonata-by-beethoven-an-analysis/> (accessed [Nov 11, 2025]).

McCormick, L. (2019). *Canonic technique in Schoenberg’s Pierrot lunaire.* LMC. <https://laurenmccormick.com.au/canonic-technique-in-schoenbergs-pierrot-lunaire> (accessed [Nov 11, 2025]).

Marks, L. E. (1982). Bright sneezes and dark coughs, loud sunlight and soft moonlight. *Journal of Experimental Psychology, 8*(2), 177–193. <https://doi.org/10.1037/0096-1523.8.2.177>

Reed, J. (2025). Kant, Coleridge, and the “Moonlight” sonata: Imagination, fantasy, and fantasies in Beethoven’s time. *19th-Century Music,* *48*(3), 116–139. <https://doi.org/10.1525/ncm.2025.48.3.116>

Symth, D. H. (1980). The music of Pierrot lunaire: An analytic approach. *Theory and Practice, 5*(1), 5–24. <http://www.jstor.org/stable/41330184>

1. According to Kerney (2017) and Dunchen (2024), the name *Moonlight Sonata* was coined by Ludwig Rellstab, for whom listening to the sonata led to imagining ‘a boat visiting, by moonlight, the primitive landscapes of Lake Lucerne’. Similarly, one of Beethoven’s students, Carl Czemy, remarked that the sonata alluded to a ‘nocturnal scene in which a mournful ghostly voice sounds from the distance’. As such, it is worth noting that Beethoven may never have composed the sonata with the moon in mind. In fact, Beethoven’s own notes refer to Mozart’s *Don Giovanni*, specifically the scene in which Don Juan kills the commander (a piece that is composed in the same key as the *Moonlight Sonata*). Consequently, music practitioners believe that Beethoven envisioned a more funeral theme (compared to the moon or romantic feel associated with music listeners) when composing the *Moonlight Sonata* (titled *Piano Sonata no. 14 in C# minor, op. 27 no. 2*). This funereal, as opposed to romantic, theme is also supported by the fact that *Moonlight Sonata* was composed around the same time as the composer’s hearing loss was becoming problematic. The conflicting visual imagery evoked for the composer versus for the listener highlights the role of visual imagery in shaping one’s emotional experience and narrative associated with a musical composition. [↑](#footnote-ref-1)
